# Reduced Incidence and Better Liver Disease Outcomes among Chronic HCV Infected Patients Who Consume Cannabis

**DOI:** 10.1155/2018/9430953

**Published:** 2018-09-23

**Authors:** Adeyinka Charles Adejumo, Oluwole Muyiwa Adegbala, Kelechi Lauretta Adejumo, Terence Ndonyi Bukong

**Affiliations:** ^1^Department of Medicine, North Shore Medical Center, Salem, MA, USA; ^2^Department of Medicine, University of Massachusetts Medical School, Worcester MA, USA; ^3^School of Public Health, University of Massachusetts Lowell, Lowell, MA, USA; ^4^Department of Medicine, Englewood Hospital and Medical Center, Englewood, NJ, USA; ^5^INRS-Institut Armand-Frappier, Institut National de la Recherche Scientifique, Laval, QC, Canada

## Abstract

**Background and Aim:**

The effect of cannabis use on chronic liver disease (CLD) from Hepatitis C Virus (HCV) infection, the most common cause of CLD, has been controversial. Here, we investigated the impact of cannabis use on the prevalence of CLD among HCV infected individuals.

**Methods:**

We analyzed hospital discharge records of adults (age ≥ 18 years) with a positive HCV diagnosis. We evaluated records from 2007 to 2014 of the Nationwide Inpatient Sample (NIS). We excluded records with other causes of chronic liver diseases (alcohol, hemochromatosis, NAFLD, PBC, HBV, etc.). Of the 188,333 records, we matched cannabis users to nonusers on 1:1 ratio (4,728:4,728), using a propensity-based matching system, with a stringent algorithm. We then used conditional regression models with generalized estimating equations to measure the adjusted prevalence rate ratio (aPRR) for having liver cirrhosis (and its complications), carcinoma, mortality, discharge disposition, and the adjusted mean ratio (aMR) of total hospital cost and length of stay (LOS) [SAS 9.4].

**Results:**

Our study revealed that cannabis users (CUs) had decreased prevalence of liver cirrhosis (aPRR: 0.81[0.72-0.91]), unfavorable discharge disposition (0.87[0.78-0.96]), and lower total health care cost ($39,642[36,220-43,387] versus $45,566[$42,244-$49,150]), compared to noncannabis users (NCUs). However, there was no difference among CUs and NCUs on the incidence of liver carcinoma (0.79[0.55-1.13]), in-hospital mortality (0.84[0.60-1.17]), and LOS (5.58[5.10-6.09] versus 5.66[5.25-6.01]). Among CUs, dependent cannabis use was associated with lower prevalence of liver cirrhosis, compared to nondependent use (0.62[0.41-0.93]).

**Conclusions:**

Our findings suggest that cannabis use is associated with decreased incidence of liver cirrhosis, but no change in mortality nor LOS among HCV patients. These novel observations warrant further molecular mechanistic studies.

## 1. Introduction

Globally chronic Hepatitis C Infection (HCV), followed by alcoholic and nonalcoholic-fatty liver diseases (ALD and NAFLD), represents the most common cause of chronic liver disease (CLD) that can progress to liver fibrosis and hepatic cellular carcinoma (HCC) [1]. In the United States of America (USA), HCV imposes significant financial and utilization burden on the healthcare system [2]. Fortunately, with recent advances in HCV therapy using direct-acting antivirals (DAA) [3], sustained virologic response (SVR) has been achieved in about 95% of infected individuals [4], resulting in gradual decline in the burden of HCV [1]. However, the remaining 5% with uncontrolled SVR might still progress to CLD. Furthermore, individuals who develop resistance to or do not have access to DAA such as individuals with modest incomes or from less developed countries are more likely to still suffer from CLD [5, 6].

Cannabis, the most commonly used illicit drug [7], has been shown to modulate inflammatory and fibrotic processes in the liver in preclinical studies [8, 9]. These preclinical observations have additionally been confirmed by recent population studies on ALD and NAFLD [10, 11]. However, the relationship between cannabis use and HCV has been controversial and remains unclear. While earlier studies suggested that cannabis use resulted in increased steatosis, fibrosis, and worsening of HCV disease [12–14], recent observations are now advancing that cannabis use has no effect on HCV disease progression [15–17]. A recent reports even revealed that cannabis use was associated with reduced steatosis in HCV infected individuals [18]. However, most of these studies had significant limitations given that evaluations were made from a single center. Further, these studies were limited in size and lacked diversity in study individuals. Additionally, these studies often included subjects with other chronic liver diseases, which might impact the effects of cannabis on HCV disease study outcomes. Interestingly, a recent study revealed that cannabidiol (CBD), the main nonpsychoactive agent in cannabis, induced cell death in approximately 85% of HCV infected cells in vitro, similar to interferon alpha-2b treatment [19].

In this study, we examined a large, nationally representative sample of over 900,000 subjects with chronic HCV infection. We used propensity-matched analysis with the objective of eliminating differences in demographics and comorbid characteristics of study participants. We additionally investigated the relationships between cannabis versus noncannabis use on the prevalence of liver cirrhosis and liver carcinoma in our study population. Furthermore, we estimated how cannabis use affected hospitalization outcomes: mortality, discharge disposition, cost, and length of stay (LOS).

Our investigations revealed that, among HCV infected individuals, cannabis use was associated with decreased prevalence of liver cirrhosis and lower overall treatment cost.

## 2. Materials and Methods

### 2.1. Study Population and Design

Our study data included hospital discharge records of adults (≥18 years old) with a diagnosis of HCV infection (HCV+). These records are comprised of hospitalized patients in the USA from 2007 to 2014, archived in the Healthcare Cost and Utilization Project Nationwide Inpatient Sample (HCUP-NIS) database. HCUP-NIS is the largest inpatient database in the USA, produced and maintained by the Agency for Healthcare Research and Quality (AHRQ) [20]. HCUP-NIS contains about 7-8 million records from over 3,000 nonfederal hospitals across more than 30 states in the US [20]. Each record has up to 30 different clinical diagnoses, encoded with the International Classification of Diseases, Ninth Edition, Clinical Modification (ICD-9-CM). Our study design is a propensity-matched cohort study. As the HCUP-NIS is a completely deidentified and the data is readily available publicly, we did not require an Institutional Review Board approval.

### 2.2. Selection of Cannabis Cohort and Propensity Matching Variables

**Figure 1:**
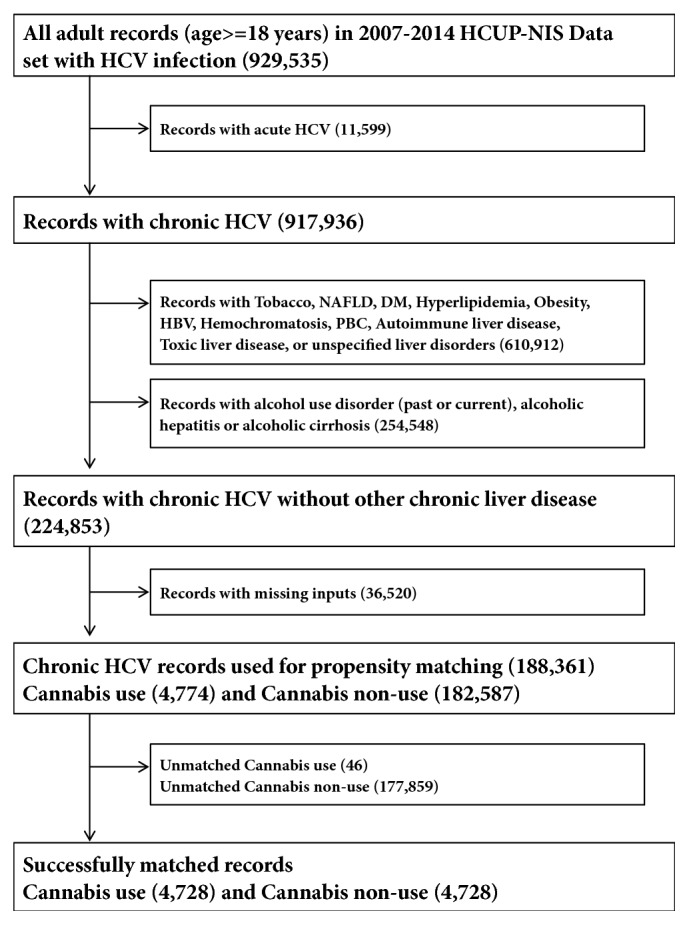
**Selection flow chart of study populations.** Illustrative flow chart of how our study population was grouped for statistical analysis to determine the impact and disease outcomes among HCV infected individuals who use cannabis.

All clinical attributes were either identified as variables (demographics, region, and hospital factors) in the dataset or retrieved from containing variables using the International Classification of Diseases, Ninth Revision, Clinical Modification (ICD-9-CM). After pooling records collected from January 1, 2007, to December 31, 2014, in the HCUP-NIS, we first identified HCV+ (acute and chronic) records and then eliminated records with acute HCV+, chronic HCV+ individuals with a history of abusive alcohol use and diagnosis of alcoholic liver diseases. We also excluded records with risk factors for CLD or those having other potential causes of CLD (tobacco use, nonalcoholic fatty liver disease, diabetes mellitus, hyperlipidemia, obesity, HBV, PBC, autoimmune liver disease, toxic liver diseases, chronic passive congestion of the liver, hepatic infarction, hepatitis from other viral illnesses, and unspecified liver disorders) (Figure 1). The ICD-9-CM codes used have been utilized to identify HCV infections and outcomes from the HCUP-NIS by numerous studies [10, 11, 21–23].

Cannabis users were identified using ICD-9-CM codes, and this code selects patients using Indian hemp, marijuana, and different varieties of cannabinoid-containing substances. We then retrieved data from the NIS on demographics features including age, gender (male, female), race (White, Black, Hispanics, and other races), primary health insurance (Medicare, Medicaid, private, self-pay, and others), income, region, and hospital teaching status. Income was stratified into four based on average household income of the zip-code of residence. Region was stratified into four based on US Census Bureau: Northeast, Midwest, South, and West. Hospital teaching status was classified into three: rural, urban nonteaching, and urban teaching.

In order to develop a logistic model to predict cannabis use, we used ICD-9-CM codes to identify various factors/comorbidities that might be associated with cannabis usage: peripheral vascular disease, chronic heart failure, hypertension, chronic lung disease, valvular heart disease, cardiac arrhythmias, chronic kidney disease, cerebral vascular disease, ischemic heart disease, hypothyroidism, hyperthyroidism, acquired immune deficiency syndrome, other substance abuse, and malignancies. Some of these variables were either identified in literature to be associated with cannabis use or included as general comorbid disorders common among inpatients.

### 2.3. Outcome Variables

After matching, we identified 13 outcomes: three primary and ten secondary. Our primary outcomes were having a diagnosis of liver cirrhosis, liver carcinoma, and in-hospital mortality. Six of our ten secondary outcomes are known complications of liver cirrhosis including ascites, variceal bleeding, hepatorenal syndrome, hepatic encephalopathy, portal hypertension, and jaundice. The seventh secondary outcome assessed the proportion of individuals with decompensated versus nondecompensated cirrhosis. We deduced cirrhotic decompensation by using the complications of cirrhosis mentioned above, to stratify patients based on the Baveno4 methodology. The three other outcomes were length of stay (LOS), total hospital cost, and disposition on discharge. All pre-2014 total hospital cost values were escalated to the 2014 numbers with the inflation rate. Unfavorable disposition on discharge was defined as discharge to secondary health facilities versus others (discharged home with/without home health care aide). Besides mortality, LOS, total hospital cost, and discharge disposition, which were variables in the dataset, all the other outcomes were identified using ICD-9-CM codes. The liver cirrhosis and the Baveno4 system codes have been extensively used and validated in the NIS [24, 25].

### 2.4. Statistical Analysis

Continuous variables were represented with a mean (standard deviation) or median (interquartile range), depending on the underlying distribution, compared with Mann-Whitney U test; categorical variables are presented with percentages, compared with Rao-Scott Chi-square.

After selecting our chronic HCV+ population, we identified cannabis users and developed a multivariate logistic regression model, with stepwise negative selection, to generate propensity scores for cannabis use versus noncannabis use. With a caliper less than 0.2 of the standard deviation of the logit of the propensity scores, we used a greedy-match algorithm (gmatch macro) to match cannabis users to nonusers in a 1:1 ratio [26]. Propensity matching has been shown to eliminate a large percentage of residual confounding in observational studies, such as ours, and to generate effect estimates that are comparable with a randomized clinical control trial in the magnitude and outcomes [27, 28]. After matching, paired equivalents of the tests above such as Paired T-test, Wilcoxon signed-rank test, and McNemar's test were used to compare differences in outcomes between cannabis users versus nonusers.

Next, we developed conditional Poisson regression models, with robust modification of the error variance, to estimate the adjusted prevalence rate ratio (adjusted by matching) [aPRR] of having liver cirrhosis, the complications of liver cirrhosis including ascites, variceal bleeding, hepatorenal syndrome, hepatic encephalopathy, portal hypertension, and jaundice, and other noncirrhotic outcomes: liver carcinoma, mortality, cost, LOS, and unfavorable discharge to secondary health facilities [29]. For the noncirrhotic outcomes, we additionally adjusted for presence of cirrhosis, to eliminate the confounding effect of cirrhosis on health care utilization, as cirrhosis has been shown to be the major pathologic pathway of liver cancer in HCV+ livers and to also contribute to a significant healthcare utilization. Among individuals with liver cirrhosis, we designed additional Poisson models for aPRR for having decompensated versus compensated cirrhosis with respect to cannabis exposure, using the Baveno4 classification system. The models were specified to match the outcomes: gamma for total hospital cost and negative binomial for LOS to generate the adjusted mean ratios (aMR).

To investigate if the amount of cannabis used had an impact on the prevalence of liver cirrhosis, we performed additional analyses. Using the ICD-9-CM codes, we categorized cannabis users into two groups (dependent and nondependent users), to loosely approximate the quantity and frequency of use, since the HCUP-NIS data does not contain such information. Then we recalculated the effect of cannabis use on cirrhosis, with Bonferroni adjustment of the confidence intervals to account for the grouping of cannabis users. Because our result revealed that the frequency of cirrhosis remained lower among cannabis users after matching, we redesigned the propensity matching factors to include liver cirrhosis, to completely eliminate a possible confounding effect of cirrhosis in the matched cohort. Then, we reestimated the health care utilization outcomes, with respect to cirrhosis. All models used a p-value of <0.05, reported with a 95% confidence intervals (CI), and were performed using Statistical Analysis System (SAS V.9.4, SAS Institute Inc., Cary, NC, US). We plotted the estimates with GraphPad Prism 7.

## 3. Results

### 3.1. Study Population before and after Matching

After eliminating records with acute HCV+, abusive alcohol use, alcoholic liver disorders, other etiologies of chronic liver diseases, and records with missing inputs, there were 4,774 records with cannabis use disorder out of a total of 188,361 records (Figure 1). Cannabis users were younger (mean age of 40 years versus 53 years for nonusers), more likely to be White, to be on Medicaid or Self-pay/Uninsured, and to be from the lower income quartiles (Table 1). Besides having higher frequency of other substance abuse and similar frequencies for hyperthyroidism and AIDS, cannabis users had lower percentages for all the other comorbidities. The frequency of the outcomes of the study including liver cirrhosis, complications of cirrhosis, mortality, liver cancer, and unfavorable discharge disposition were all lower among patients with cannabis use (Table S1).

During propensity matching, 4,728 of 4,774 cannabis users were successfully matched to an equal number of nonusers (Table S2). The frequencies of demographic and comorbid characteristics became statistically identical among the matched cannabis versus noncannabis users. Furthermore, matching eliminated the aOR for having cannabis use disorder with respect to different factors (Table S3).

### 3.2. Outcomes among Propensity-Matched Cohort

**Figure 2:**
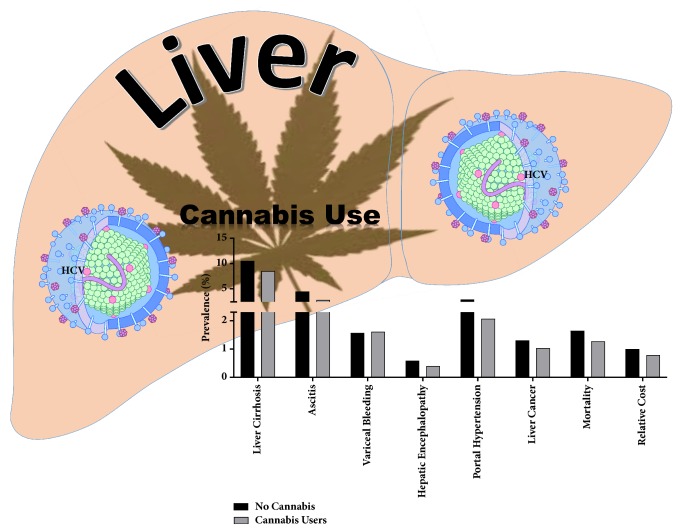
**Summary schematic of the effects of cannabis use by HCV infected individuals and liver disease associated parameters.** Our observations suggest that cannabis use by HCV infected individuals is associated with significant reduction in adverse progressive disease associated pathologies. Further, cannabis users had an overall lower cost of disease management compared to noncannabis users. Schematic illustrations made use of some motifolio templates (http://www.motifolio.com/).

After matching, cannabis users had lower frequencies for liver cirrhosis and its complications, lower frequencies of higher Baveno4 scores, and unfavorable discharges. But the frequency of mortality and liver cancer were similar between cannabis users and nonusers (Table 2). On regression analysis (Table 3 and Figure 2), when compared to nonusers, cannabis users had lower prevalence rate ratio for liver cirrhosis (aPRR:0.81[0.72-0.91]). They had a cirrhosis prevalence rate of 40.18 per 1,000 hospitalizations versus 49.72 per 1,000 hospitalizations among nonusers (Table 4). Furthermore, cannabis users had decreased frequencies of ascites and portal hypertension, while other complications of cirrhosis were similar across noncannabis users. The adjusted prevalence of liver cancer and in-hospital mortality was similar across both cannabis and noncannabis user groups. While the LOS was similar among both groups (5.57[5.10-6.09] versus 5.66[5.25-6.10] days), cannabis users had decreased total hospital cost ($39,642[36,220-43,387] versus $45,566[$42,244-$49,150]) and less unfavorable discharge disposition (0.87[0.78-0.96]).

### 3.3. Post Hoc Analyses

After categorizing cannabis users into two groups (dependent and nondependent users), we found that the prevalence of liver cirrhosis decreased by 15% and 48% among nondependent (0.85[0.73-0.99], p-value: 0.0272) and dependent users (0.52[0.35-0.79], p-value: 0.0004), respectively. Further, compared to nondependent cannabis users, dependent users had a 38% decreased prevalence of liver cirrhosis (0.62[0.41-0.93], p-value: 0.0156).

After redesigning the propensity matching with cirrhosis as a predictor of cannabis use, the associations between cannabis use and healthcare utilization was similar to the previous model, which did not match with cirrhosis, but adjusted for cirrhosis as a predictor (Table S4).

## 4. Discussion

Our study utilizing a large dataset spanning records from 2007 to 2014 of NIS revealed novel associations between cannabis use and chronic liver disease among HCV infected individuals. Using propensity matching to effectively eliminate many confounding differences, our studies revealed that cannabis use was associated with more favorable HCV infection outcomes. Compared to other population studies performed before 2010, which all suggested that cannabis use resulted in steatosis and cirrhosis among HCV [12–14], our results revealed the contrary. These three earlier studies had limited sample sizes (204, 270, and 315 subjects) and included patients with alcohol and tobacco use, which might have resulted in a different outcome compared to our study.

Cannabinoids act through CB-1 and CB-2 receptors, with pro- and antifibrotic effects respectively. Early studies mostly rationalized with the fact that HCV was associated with a significant induction in hepatic CB-1 receptor expression [30]. However, later studies between 2013 and 2016 among HCV [16] or HCV+HIV [15, 17] coinfected individuals concluded that cannabis had no effect on liver disease from HCV. Although these three newer studies had larger samples sizes (550, 575, and 690 subjects), they did not eliminate many chronic liver diseases from diverse etiologies. Further, a more recent study on HCV+HIV coinfected patients in 2017 [18] used an even larger sample size (838 subjects) and revealed that cannabis use was associated with a decrease in hepatic steatosis. Our analysis extended on these previous studies in an attempt to overcome limitations of sample size and inclusions of liver disease from non-HCV infection and other synergistic etiologies. We also excluded other causes of CLD and used a more recent nationally representative dataset. Further, we additionally investigated a larger array of clinical outcomes and health care utilization indicators.

Cannabinoids have been demonstrated to kill profibrotic hepatic stellate cells [31] and reverse liver fibrosis/cirrhosis in preclinical models after chronic administration [32]. These actions might explain our findings of decreased hepatic cirrhosis and its attendant complications, which are all secondary to portal hypertension from cirrhosis. Likewise, cannabigerol, a nonpsychotropic component of cannabis thwarts the growth of colorectal cancer cells [33], and tetrahydrocannabinol inhibited glioblastoma cell growth both in vitro and in patients [34]. Both of these mechanisms and decreased prevalence of cirrhosis might explain why cannabis users had lower prevalence of liver carcinoma.

We reveal that cannabis was associated with reduced unfavorable discharge and hospital cost. These outcomes might be due to less burden of CLD because of the smaller frequency of cirrhosis and carcinoma. Cannabis has been shown to be related to lower mortality among hospitalized cancer patients [35].

In summary, the effect of cannabis on HCV disease might be multifaceted. First, cannabis might be directly toxic to hepatitis virus in vivo, as is recently shown in vitro [19]. Second, cannabis users might make HCV patients feel less nauseous and more motivated to take their other antiviral medications and another medical regimen [36]. Third, cannabis might be associated with decreased hepatic cirrhosis and complications of cirrhosis, thereby resulting in the lower cost and better discharge disposition outcomes among cannabis users. Thee potential roles played by cannabis use on liver disease progression in HCV positive patients will require additional complementary evaluation by way of prospective studies.

### 4.1. Study Strengths and Limitations

The major weaknesses in our study are the cross-sectional design, recall biases, coding errors in the ICD-9-CM application, lack of information on medications such as antiviral therapies, type of cannabis ingested, mode of cannabis use (oral versus inhalation), and sensitivity and specificity of ICD-9-CM coding for cannabis use disorder. Absence of data on which patients received the new direct-acting antiviral therapy is a significant limitation, given that these medications are extremely effective and significantly modulate the progress of HCV liver disease. We mitigated many of these errors by first eliminating other causes of CLD and using a propensity-matched analysis, which directly balanced and eliminated the effect of factors associated with cannabis use in the NIS and allowed a less biased estimation of our outcomes. However, it is possible that additional unmeasured confounding factors might still impact our observations. These errors are likely to similarly impact both cannabis and noncannabis use groups and would likely decrease our observed effects on cannabis use and the incidence of CLD. Therefore, the true effect of cannabis use on HCV might be stronger than observed estimates in our current study. Furthermore, the large size of the NIS data allowed us to conduct a very efficient propensity matching and to capture subjects from all over the USA, making our result more generalizable.

## 5. Conclusions

Our novel study to the best of our knowledge represents the largest population-based study to assess the effect of cannabis use on CLD associated with HCV infection. Our observations suggest cannabis use might have a positive impact in alleviating complications of portal hypertension, liver cancer, and disease outcomes among HCV infected individuals. These novel findings require additional prospective and translational molecular research studies to decipher which specific active components of cannabis impact liver disease development during chronic HCV infection.

## Figures and Tables

**Table 1:** Baseline characteristics of chronic hepatitis C infected patients, by cannabis use status (before propensity matching).

	**No Cannabis use**	**Cannabis use**	**p-value**
n (~weighted)	182587 (~887,975)	4774 (~23,371)	

Age, mean (SD)	53.06 (14.15)	40 (12.96)	<0.0001

Gender			0.3599
Male	53.86	54.60	
Female	46.14	45.40	

Race			<0.0001
White	59.90	66.23	
Black	21.63	18.92	
Hispanics	12.74	10.73	
Others	5.74	4.12	

Health insurance			<0.0001
Medicare	33.91	20.92	
Medicaid	30.91	46.10	
Private	21.00	12.05	
Self-pay & others	14.18	20.93	

Income			<0.0001
Lowest Quartile	38.04	45.92	
Second Quartile	24.42	25.41	
Third Quartile	20.99	18.50	
Highest Quartile	16.55	10.17	

Hospital region			<0.0001
Northeast	24.99	22.23	
Midwest	11.72	15.18	
South	39.69	39.21	
West	23.60	23.38	

Hospital teaching status			<0.0001
Rural	7.06	10.35	
Urban non-teaching	32.71	33.34	
Urban teaching	60.23	56.31	

Peripheral vascular disease	2.43	1.05	<0.0001

Congestive heart failure	6.23	2.59	<0.0001

Hypertension	35.92	22.34	<0.0001

Chronic lung disease	16.49	16.21	0.6303

Valvular heart disease	3.14	1.74	<0.0001

Cardiac arrhythmias	10.24	5.29	<0.0001

Chronic kidney disease	12.64	4.49	<0.0001

Cerebral vascular disease	3.45	1.71	<0.0001

Ischemic heart disease	8.68	4.77	<0.0001

Hypothyroidism	7.32	3.65	<0.0001

Hyperthyroidism	0.38	0.38	0.9553

Other substance abuse	15.83	65.38	<0.0001

Malignancies	9.95	3.55	<0.0001

AIDS	2.89	2.59	0.2394

**Table 2:** Outcome characteristics by cannabis use status, after propensity matching.

	**No Cannabis use**	**Cannabis use**	**p-value**
n	4728	4728	

Liver cirrhosis	10.55	8.52	0.0014

Ascites	4.51	2.79	<0.0001

Variceal bleeding	1.57	1.61	0.0258

Hepatorenal syndrome	0.49	0.38	0.4329

Hepatic encephalopathy	0.59	0.40	0.1964

Portal hypertension	2.92	2.07	0.0108

Jaundice	0.53	0.34	0.158

Baveno4 scoring			0.0019
0: No cirrhosis	89.45	91.48	
1: Compensated cirrhosis	5.31	4.72	
2: Decompensated cirrhosis	5.25	3.81	

Mortality	1.65	1.27	0.119

Liver cancer	1.31	1.04	0.2282

Discharge disposition			0.0014
Favourable discharge	80.71	83.27	
Unfavourable discharge	19.29	16.73	

**Table 3:** Comparison of liver disease and outcomes among HCV patients.

	**aPRR/aMR**	**LCL**	**UCL**	**p-value**
Liver cirrhosis	0.81	0.72	0.91	0.0004
Liver cancer*∗*	0.79	0.55	1.13	0.2013
**Complications of cirrhosis**				
Ascites	0.62	0.50	0.76	<0.0001
Variceal bleeding	1.03	0.75	1.40	0.8658
Hepatorenal syndrome	0.78	0.42	1.45	0.436
Hepatic encephalopathy	0.68	0.38	1.20	0.1824
Portal hypertension	0.71	0.56	0.91	0.0065
Jaundice	0.64	0.34	1.20	0.1633
Baveno4	0.97	0.93	1.02	0.2167
**Health care utilization**				
Inpatient mortality*∗*	0.84	0.60	1.17	0.298
Length of stay*∗*	0.99	0.93	1.05	0.6319
Total hospital cost*∗*	0.87	0.80	0.95	0.0012
Unfavourable discharge*∗*	0.85	0.77	0.95	0.0025

aPRR: adjusted prevalence rate ratio; aMR: adjusted mean ratio (LOS and total hospital cost). LCL and UCL: lower and upper confidence limit; *∗*: effects are after adjusting for cirrhosis.

**Table 4:** Adjusted estimates of liver disease, mortality, and outcomes of HCV patients.

	**Cannabis use**			**No Cannabis use**		

	**Prevalence**	**LCL**	**UCL**	**Prevalence**	**LCL**	**UCL**

Cirrhosis	54.8	48.29	62.18	67.85	60.66	75.89
Cancer*∗*	3.265	1.819	5.859	4.131	2.356	7.243
Mortality*∗*	1.741	1.194	2.536	2.078	1.486	2.907

	**Mean**	**LCL**	**UCL**	**Mean**	**LCL**	**UCL**

LOS, days	5.5762	5.104	6.092	5.6611	5.251	6.103
Charge, $	39642	36220	43387	45566	42244	49150

	**Percentage**	**LCL**	**UCL**	**Percentage**	**LCL**	**UCL**

Unfavourable discharge	25.18	22.28	28.46	29.57	26.3	33.26

LCL and UCL: lower and upper confidence limit.

## Data Availability

The relevant data can be accessed at the following link: https://www.hcupus.ahrq.gov/nisoverview.jsp.

## Abbreviations

AC: Alcoholic cirrhosis
AH: alcoholic hepatitis
ALD: Alcoholic liver disease
AS: Alcoholic steatosis
CBD: Cannabidiol
CLD: Chronic liver disease
CU: Cannabis use
HBV: Hepatitis b viral infection
HCC: Hepatocellular carcinoma
HCV: Hepatitis c viral infection
THC: Tetrahydrocannabinol.

## Supplementary Materials

Supplementary Materials Table S1: outcome characteristics by cannabis use status, before propensity matching. Table S2: baseline characteristics of chronic hepatitis C infected patients, by cannabis use status (after propensity matching). Table S3: odds ratio of having cannabis use disorder, before and after propensity matching. Table S4: comparison of liver disease and outcomes among HCV patients, after including cirrhosis as match predictor.
