# Supplementary material for: Reduced Incidence and Better Liver Disease Outcomes among Chronic HCV Infected Patients Who Consume Cannabis
Source: Can J Gastroenterol Hepatol. 2018 Sep 23;2018:9430953. doi: 10.1155/2018/9430953 (PMC6174743; doi:10.1155/2018/9430953)
Supplement: Supplementary Materials — Table S1: outcome characteristics by cannabis use status, before propensity matching. Table S2: baseline characteristics of chronic hepatitis C infected patients, by cannabis use status (after propensity matching). Table S3: odds ratio of having cannabis use disorder, before and after propensity matching. Table S4: comparison of liver disease and outcomes among HCV patients, after including cirrhosis as match predictor. [file 9430953.f1.pdf]

Supplementary tables

Table S1

n	**No Cannabis use** 182587 (~887,975)	**Cannabis use** 4774 (~23,371)	**p-value**
Liver cirrhosis	23.73	8.49	<0.0001
Ascites	11.25	2.77	<0.0001
Variceal bleeding	4.56	1.58	<0.0001
Hepatorenal syndrome	1.48	0.38	<0.0001
Hepatic encephalopathy	1.45	0.40	<0.0001
Portal hypertension	7.03	2.11	<0.0001
Jaundice	0.83	0.32	<0.0001
Baveno 4 scoring			<0.0001
0: No cirrhosis	76.27	91.51	
1: Compensated cirrhosis	10.60	4.67	
2: Decompensated cirrhosis	13.13	3.82	
Mortality	3.93	0.03	<0.0001
Liver cancer	4.54	1.04	<0.0001
Discharge disposition			<0.0001
Favourable discharge	77.12	83.23	
Unfavourable discharge	22.88	16.77	

Table S1: Outcome characteristics by cannabis use status, before propensity matching

Table S2

	No Cannabis use	Cannabis use	p-value
n (~weighted)	4728	4728	
Age, mean (SD)	40.41 (13.12)	40.62 (12.98)	0.4678
Gender			0.3784
Male	53.72	54.72	
Female	46.28	45.28	
Race			0.974
White	66.54	66.46	
Black	19.37	19.14	
Hispanics	10.11	10.38	
Others	3.98	4.02	
Health insurance			0.461
Medicare	19.75	21.04	
Medicaid	47.08	45.94	
Private	12.56	12.14	
Self-pay & others	20.60	20.88	
Income			0.4098
Lowest Quartile	47.19	45.71	
Second Quartile	25.51	25.34	
Third Quartile	17.58	18.65	
Highest Quartile	9.73	10.30	
Hospital region			0.9886
NorthEast	10.26	10.30	
Midwest	33.23	33.35	
South	56.51	56.35	
West			
Hospital teaching status	22.97	22.46	0.4856
Rural	14.26	14.89	
Urban non-teaching	40.84	39.68	
Urban teaching	21.93	22.97	
Peripheral vascular disease	0.82	1.06	0.2444
Congestive heart failure	2.56	2.60	0.8976
Hypertension	21.85	22.34	0.5962
Chronic lung disease	15.61	16.22	0.4383
Valvular heart disease	1.99	1.73	0.3594
Cardiac arrhtymias	4.67	5.22	0.2312
Chronic kidney disease	4.12	4.51	0.3869
Cerebral vascular disease	2.09	1.73	0.2052
Ischemic heart disease	4.95	4.78	0.7077

Hypothyroidism	3.68	3.66	0.9575
Hyperthyroidism	0.53	0.38	0.2946
Other substance abuse	65.38	65.27	0.9369
Malignancies	3.17	3.57	0.3052
AIDS	2.92	2.60	0.3653

Table S2: Baseline characteristics of chronic hepatitis C infected patients, by cannabis use status (after propensity matching)

Table S3

	**Before propensity matching**				**After propensity matching**			
	**aOR**	**LCL**	**UCL**	**p-value**	**aOR**	**LCL**	**UCL**	**p-value**
**Age**	0.96	0.96	0.96	<0.0001	1.00	0.99	1.00	0.6176
**Female vs. Male**	0.63	0.59	0.68	<0.0001	0.97	0.88	1.07	0.4998
**Race**				<0.0001				0.9561
Black vs. White	1.23	1.11	1.37		0.99	0.88	1.13	
Hispanics vs. Whites	0.79	0.70	0.89		1.04	0.89	1.22	
Others vs . Whites	0.94	0.80	1.11		1.02	0.82	1.27	
**Health insurance**				<0.0001				0.472
Medicare vs. Private	1.47	1.31	1.64		1.12	0.95	1.31	
Medicaid vs. Private	1.25	1.12	1.39		1.02	0.89	1.18	
Self-pay & others vs. Private	1.25	1.10	1.43		1.06	0.90	1.24	
**Income status**				<0.0001				0.4251
Lowest vs. highest quartile	1.46	1.29	1.65		0.91	0.77	1.06	
Second vs. highest quartile	1.34	1.18	1.52		0.93	0.79	1.10	
Third vs. highest quartile	1.24	1.09	1.41		0.99	0.84	1.18	
**Hospital teaching status**				<0.0001				0.8936
Urban non-teaching vs. rural	0.84	0.72	0.98		0.96	0.82	1.13	
Urban teaching vs. rural	0.69	0.60	0.81		0.97	0.83	1.14	
**Hospital region**				<0.0001				0.6246
Midwest vs. NorthEast	1.48	1.29	1.71		1.08	0.93	1.25	
South vs. NorthEast	1.32	1.15	1.51		1.01	0.89	1.14	
West vs. NorthEast	1.60	1.42	1.81		1.06	0.93	1.21	
**Comorbidities**								
Valvular heart disease	0.82	0.64	1.05	0.1136	0.86	0.64	1.16	0.32
Ischemic heart disease	1.09	0.93	1.27	0.2976	0.93	0.76	1.13	0.4678
Chronic kidney disease	0.58	0.50	0.67	<0.0001	1.07	0.86	1.34	0.5312
Cardiac arrhtymias	0.90	0.79	1.04	0.1591	1.12	0.92	1.36	0.2524
Chronic lung disease	1.18	1.08	1.29	0.0003	1.05	0.93	1.18	0.4258

Hypertension	1.04	0.96	1.13	0.3545	1.02	0.91	1.15	0.6856
Congestive heart failure	0.75	0.62	0.90	0.0024	1.01	0.77	1.31	0.9586
Cerebral vascular disease	0.83	0.66	1.04	0.11	0.82	0.61	1.11	0.1939
Peripheral vascular disease	0.90	0.67	1.20	0.4674	1.26	0.82	1.94	0.2944
Hypothyroidism	0.97	0.83	1.14	0.729	0.98	0.78	1.22	0.8417
Hyperthyroidism	1.15	0.71	1.88	0.572	0.69	0.37	1.30	0.2487
AIDS	0.88	0.73	1.07	0.2044	0.88	0.68	1.14	0.3393
Substance abuse	6.75	6.20	7.35	<0.0001	1.01	0.90	1.14	0.8594
Malignancies	0.72	0.61	0.86	0.0001	1.13	0.89	1.43	0.3241
Year	1.10	1.08	1.13	<0.0001	1.00	0.98	1.02	0.8109

Table S3: Odds ratio of having cannabis use disorder, before and after propensity matching

Footnotes: aOR: adjusted odds ratio, LCL & UCL: lower and upper confidence limit.

Table S4

	aPRR/aMR	LCL	UCL	p-value
Liver cancer	0.97	0.66	1.4226	0.8613
Inpatient mortality	0.95	0.67	1.3459	0.7748
Length of stay	1.01	0.95	1.0733	0.7102
Total hospital cost	0.91	0.83	0.9868	0.0231
Unfavourable discharge	0.87	0.78	0.9593	0.0060

Table S4: Comparison of liver disease and outcomes among HCV patients, after including cirrhosis as match predictor
